# Housing Management of Male Dromedaries during the Rut Season: Effects of Social Contact between Males and Movement Control on Sexual Behavior, Blood Metabolites and Hormonal Balance

**DOI:** 10.3390/ani10091621

**Published:** 2020-09-10

**Authors:** Ramadan D. EL-Shoukary, Nani Nasreldin, Ahmed S. Osman, Nesrein M. Hashem, Islam M. Saadeldin, Ayman A. Swelum

**Affiliations:** 1 Department of Animal Hygiene, Faculty of Veterinary medicine, New Valley University, El- Kharga 72511, Egypt; ramadan.dardeer@aun.edu.eg; 2 Department of Clinical pathology, Faculty of Veterinary medicine, New Valley University, El- Kharga 72511, Egypt; nany_nasr_1@vetnv.au.edu.eg; 3 Department of Biochemistry, Faculty of Veterinary Medicine, Sohag University, Sohag 82749, Egypt; ahmed_elsayed@vet.sohag.edu.eg; 4 Animal and Fish Production Department, Faculty of Agriculture (El-Shatby), Alexandria University, Alexandria 21545, Egypt; nesreen.hashem@alexu.edu.eg; 5 Department of Animal Production, College of Food and Agriculture Sciences, King Saud University, P.O. Box 2460, Riyadh 11451, Saudi Arabia; isaadeldin@ksu.edu.sa; 6 Department of Physiology, Faculty of Veterinary Medicine, Zagazig University, Zagazig 44511, Egypt; 7 Department of Theriogenology, Faculty of Veterinary Medicine, Zagazig University, Zagazig 44511, Egypt

**Keywords:** dromedary, male, behavior, breeding system, welfare, breeding

## Abstract

**Simple Summary:**

The effect of different housing management systems on the behavior, metabolites and hormonal balance in male dromedaries during the rutting season was evaluated. Male dromedaries that were housed in groups and allowed to walk around had significantly higher frequencies of ruminating, standing, walking and sexual-related behaviors than those housed individually or tied. Movement control had significant effects on blood serum metabolites and hormone concentrations. Fenced and exercised male dromedaries had higher (*p* < 0.05) concentrations of blood serum transaminases, free radicals, glucose, cholesterol and testosterone and lower (*p* < 0.05) concentrations of cortisol and triiodothyronine (T3) than tied male dromedaries. Animal social interaction is of particular importance for maintaining physical; psychological and sexual behaviors. Allowing walking-around exercise for captive animals improves their metabolic status and decreases captive stress effects. Housing systems that guarantee social interaction and physical activity are being the most suitable housing management systems for male dromedaries during the rutting season.

**Abstract:**

The current study aimed to evaluate the effect of different housing management systems on behavior, blood metabolites and hormonal balance in male dromedaries during the rutting season. Forty-eight adult male dromedaries were stratified in a two by three factorial experiment design, testing effects of social contact (single and group) and movement control (tied, fenced and exercise). During a ten-week experimental period, male dromedaries were filmed weekly for 20 min three times per day to evaluate their behaviors. Blood samples were collected and analyzed for serum metabolites and hormones. Both animal social contact and movement control significantly affected maintenance, posture and sexual behaviors. Male dromedaries housed in groups expressed higher frequencies of sexual desire-related behaviors (teeth grinding, open legs, tail flapping, dulla “soft palate exteriorization”, blathering and urination) than those individually housed. Animal movement control significantly affected sexual behavior; fenced and exercised male dromedaries expressed higher frequencies of sexual desire-related behaviors than tied ones. Male dromedaries housed in groups and allowed to walk around had significantly higher frequencies of ruminating, standing, walking and sexual-related behaviors than those housed individually or tied. Movement control had significant effects on blood serum metabolites and hormone concentrations. Fenced and exercised male dromedaries had higher (*p* < 0.05) concentrations of blood serum transaminases, free radicals, glucose, cholesterol and testosterone (2.91 and 2.09 ng/mL, respectively) and lower (*p* < 0.05) concentrations of cortisol (44.12 and 53.52 nmol/L, respectively) and triiodothyronine (1.68 and 1.91 ng/mL, respectively) than tied male dromedaries. In conclusion, animal social interaction is of particular importance for maintaining physical, psychological and sexual behaviors. Allowing walking-around exercise for captive animals improves their metabolic status and decreases captive stress effects. Housing systems that guarantee social interaction and physical activity are the most suitable housing management systems for captive male dromedaries during the rutting season.

## 1. Introduction

Global climate changes, which are characterized by increasing desertification, high temperatures and drought, provide a driver to reconsider animal species that are bred in the livestock sector [1]. Animal species that can genetically adapt to such environmental conditions should be given more attentions. Dromedary camel (*Camelus dromedarius*) is one of the most adapted animals to harsh environmental conditions [2]. In many drought- prone arid and semi-arid regions, dromedaries play a great role in the livelihood as breeding of other animals under such conditions face several obstacles. Camel breeding in intensive farming systems has been increasingly established, since reconstitution of animal species used for meat and milk production has become a crucial demand under current drastic environmental changes. Compared to other livestock species, dromedaries have received less attention by the scientific community. Thus, understanding the physiological and behavioral properties of dromedaries facilitates ideal harness of their natural advantages under intensive farming systems.

Dromedaries are classified as seasonal breeders, with a restricted breeding season that varies from two to six months [3]. The breeding season extends from December to March in Egypt [4], Tunisia [5], Somaliland [6], Pakistan [7], India [8] and Sudan [9]. In Saudi Arabia and United Arab Emirates, the breeding season extends from October to April [10,11]. This relatively short breeding season limits reproductive performance and is considered one of the most constraints facing camel breeding [12,13,14]. Moreover, during the breeding season, male dromedaries exhibit aggressive behavior and substantial physiological, morphologic and behavioral changes negatively affecting animal performance [15]. In intensive farming systems, dromedaries are kept in captive conditions. Moreover, during the rutting season, male dromedaries are conventionally kept tied with ropes in small pens and/or kept in single stalls to avoid aggression toward other males and humans [16]. This breeding system may negatively affect animal welfare and increases negative effects of sexual activity-related peculiarities on animal performance. Padalino et al. [17] stated that male dromedaries showed both oral and locomotor stereotypy most frequently when the bulls were housed in single boxes for 24 h, indicating poorer animal welfare. In context, Fatnassi et al. [15] found that male dromedaries housed in a single stall for 22 h and 30 min, with one hour of paddock time and 30 min of exposure to a female camel herd had significantly higher sexual behavior scores, behavioral repertoires and decreased cortisol levels than those housed in a single stall for 24 h and those housed in a single stall for two to three hours with one hour free in the paddock. Skidmore [18] reports that male dromedaries kept in herds tended to start breeding earlier with longer rutting periods than that of confined males, confirming the relationship between housing system and sexual behavior. Farsi et al. [19] developed a highly reliable and sufficiently accurate scoring method to assess conveniently the locomotor activity rhythm and specific behaviors in camels. Accordingly, achieving the ideal housing system of male dromedaries during the rutting season can contribute to maximizing animal reproductive performance and reducing sexual–associated aggressiveness, thus improving animal welfare. This study was designed to investigate the effect of different housing system managements considering both social contact and movement control on the maintenance, posture and sexual behaviors as well as blood metabolites and hormonal balance of captive male dromedaries during the rutting season.

## 2. Material and Methods

### 2.1. Animal and Management:

Forty-eight mature fertile male dromedary camels aged 6–9 years and weighing 400–450 kg with body condition score averaging 3.5 ± 0.35 arbitrary units (0—extremely thin, 5—obese) were enrolled in the current study [20]. The present study was conducted in the Assiut governorate (latitude 24°48′ N and longitude 46°31′ E) of Egypt and lasted for 14 weeks (4 weeks for adaptation and 10 weeks for experiment) during the rutting season (December–March). All dromedaries were housed in semi-covered area (15 m^2^/camel) with sand floors [15]. Dromedaries were fed approximately 3% of their live weight as dry matter. The diet consisted of 40% concentrate (11% protein) and 60% hay. The concentrate diet was divided into two equal weights and offered twice daily in the camel stall at 8–9 a.m. and 4–5 p.m.; hay was offered at 11 a.m.–12 p.m. Feeding quantity and quality remained constant throughout the experiment time. All animals had free access to fresh water. The experimental protocol regarding the care and management of the camels was approved by the Ethics Committee of the New Valley University, Egypt.

### 2.2. Experimental Design

The forty-eight male dromedaries were divided into two main groups according to their social contact: single housing (n = 12) or group housing (n = 36). In the single housing, each unit was 15 m^2^ (5 × 3 m) and had only single dromedary and the social contact between animals was prevented (four units of single camel per unit/group; n = 4/group). In the group housing, each semi-covered unit was 45 m^2^ (7.5 × 6 m) and had three dromedaries and the social contact between males was allowed (four units of three dromedaries per unit/group; n = 12/group).

These male dromedaries were further divided according to movement control into three groups: tied, fenced and exercised (n = 16/group). In the tied group, dromedaries were restrained with a 3.09 m rope started from the head to the manger which allowed their movement (standing, laying-down and walking) in a half circle area (15 m^2^/camel). In the fenced group, dromedaries were left free in fenced area (15 m^2^/camel). In the exercised group, male dromedaries were left unconfined in semi-covered pen having the same dimensions of pens of fenced group. However, these dromedaries had 6 km of daily walking exercise in the morning for about 20 min.

According to the interaction between the effect of social contact and movement control, six groups were formed as following: single-tied (n = 4), single-fenced (n = 4), single-exercised (n = 4), group-tied (n = 12), group-fenced (n = 12) and group-exercised (n = 12). The presence of females was completely prevented throughout the experimental period. Additionally, these experimental males were not allowed to see or contact any female—even during exercise—to avoid aggressive and fighting behavior between males.

### 2.3. Evaluation of the Behavioral Variables

Before the start of experiments, animals were housed under the same condition of the experiment for four weeks, serving as adaptation. During first few days of this period, some aggressive behaviors were noted between group-housed male dromedaries (3/unit). In this, one male camel in each unit of group-housed dromedaries showed increasingly dominant behavior while other two male dromedaries showed subdominant behavior.

Then, during experimental period, the animals were observed for one hour weekly for ten weeks during the breeding season divided into 3 phases (20 min/phase) in the morning (5:00–5:20 a.m.), at noon (12:00–12:20 p.m.) and in the afternoon (3:00–3:20 p.m.). Totally, 3600 min filmed video for the six treatment groups (600 min/group) were recorded. These videos were analyzed by scan-sampling technique; behaviors potentially lasting more than one minute were categorized as behavioral states and their duration was noted (in seconds/20 min), while pinpoint behaviors were categorized as events and their frequency was noted (n/20 min) to obtain the average of frequency and duration of each previously mentioned ethogram item (Table 1).

### 2.4. Blood Samples Collection and Biochemical Analysis

During blood sampling, animals were restrained gently only with halter and rope by an expert handler, who stroked the animals during the procedure. Blood samples were collected from all animals at the end of experiment at 9–11 a.m. via the jugular vein into sterile non-heparinized tubes. The samples were centrifuged at 3000 rpm for 10 min within 2 h of collection to separate sera and stored at −20 °C until analysis. Total serum proteins, albumin, glucose, cholesterol, inorganic phosphorus and calcium were assayed using a commercial kit (Spectrum Diagnostics, Obour, Egypt). However, glucose and cholesterol were determined using commercial test kits (Spinreact, Girona, Spain). Serum globulin was calculated by subtracting serum total protein values from corresponding albumin values. In addition, concentration of aspartate aminotransferase (AST) and alanine aminotransferase (ALT) were determined as liver function indicators, while creatinine and urea as kidney function indicators. Redox status was evaluated by determining concentrations of blood serum total antioxidant capacity (TAC), malondialdehyde (MDA) and nitric oxide (NO) using commercial kits (Spectrum Diagnostics, Obour, Egypt). All blood serum chemical analyses were assayed using a digital vis/UV spectrophotometer (Cecil instruments, Cambridge, England, Series NO. 52.232). Concentrations of blood serum cortisol, testosterone and triiodothyronine (T3) were assayed by a solid phase enzyme immunoassay (stat fax-2100, Awareness technology, INC, USA) using commercial ELISA kits (Bio Tina GmbH, Bugweg 53, 58119 Hagen, Germany).

### 2.5. Statistical Analysis

The normality of data distribution was evaluated in the Shapiro-Wilk W test. Effects of social contact between animals, movement control during housing and their interactions on different behavioral and biochemical variables were evaluated using two-way analysis of variance (ANOVA; SAS Institute, Cary, NC, USA, 2000). Social contact between animals (single versus group), movement control (tied, fenced, exercised) and the interaction between the two effects were introduced as fixed effects, using the following statistical model:

yijk = µ + Ti + Cj + (TC)ij + eijk

in which, yijk is the observed value of the dependent variable, µ is the overall mean, Ti is the fixed effect of the ith social contact between animals, Cj is the fixed effect of the jth movement control, (T × C)ij is the interaction between the two factors and eijk is the residual error. Differences among treatment group means were tested by Duncan’s new multiple range test and are considered significant at *p* < 0.05 level. All the results were expressed as the mean ± standard error of mean (SEM).

## 3. Results

### 3.1. Effect on Behavioral Patterns

The effects of social contact (single or group), movement control (tied, fenced or exercised) and their interaction on frequency and duration of maintenance and posture behaviors of male dromedaries during the rutting season are presented in Table 2. Social contact with other animals significantly affected maintenance and posture behaviors. Group-housed male dromedaries expressed higher (*p* < 0.001) frequencies of maintenance (ruminating) and posture (lying, standing and walking) behaviors than single-housed male dromedaries. Group-housed male dromedaries devoted longer times (*p* < 0.001) for ruminating and walking than single-housed male dromedaries, whereas single-housed male dromedaries devoted longer times (*p* < 0.001) for lying and standing behaviors. Movement-control method significantly affected maintenance and posture behaviors. Compared to tied and fenced male dromedaries, exercised male dromedaries expressed higher frequencies and longer durations (*p* < 0.001) of ruminating, standing and walking behaviors. On the other hand, tied male dromedaries gave preference (*p* < 0.001) to lying behavior compared to fenced and exercised male dromedaries. Analysis of animal social contact by movement control showed that, regardless of social contact, male dromedaries that group housed in contact with other dromedaries, expressed higher frequencies and longer durations (*p* < 0.001) of ruminating than male dromedaries in other housing systems. Male dromedaries in the tied housing system expressed higher frequencies and longer durations (*p* < 0.001) of lying behavior than male dromedaries in other housing systems. Male dromedaries that were fenced or were exercised (single-fenced, single-exercised, group-fenced and group-exercised) expressed higher frequencies and longer durations (*p* < 0.001) of standing and walking behaviors than male dromedaries in other housing systems.

### 3.2. Effect on Sexual Activity

The effects of animal social contact (single or group), movement control (tied, fenced or exercised) and their interaction on sexual behavior of male dromedaries during the rutting season are presented in Table 3. Animal social contact significantly affected the sexual behavior of male dromedaries. Male dromedaries housed in groups expressed higher frequencies of sexual desire-related behaviors (teeth grinding, opening legs, flapping tail, extruding dulla, blathering and urinating) than those individually housed. Movement control significantly affected sexual behavior; fenced and fenced exercised male dromedaries expressed higher frequencies of sexual desire-related behaviors than tied male dromedaries. Animal social contact by movement control interaction revealed that male dromedaries that controlled by tying whether in contact with other animals or in groups single-tied and group-tied expressed lower (*p* < 0.05) sexual desire-related behaviors than camel in other housing systems.

### 3.3. Effect on Blood Serum Major Proteins and Energy-Yielding Metabolites

The effects of animal social contact (single or group), movement control (tied, fenced or exercised) and their interaction on concentrations of blood serum major proteins and energy-yielding metabolites of male dromedaries during the rutting season are presented in Table 4. Animal social contact did not affect the concentrations of blood serum major proteins and energy-yielding metabolites (*p* > 0.05). Movement-control method significantly affected concentrations of blood serum major proteins, glucose and cholesterol. Tied male dromedaries had the highest (*p* < 0.001) concentrations of blood serum total protein, followed by exercised and fenced male dromedaries. Tied male dromedaries had the highest (*p* < 0.001) globulin concentrations compared to exercised and fenced male dromedaries, whereas concentrations of blood serum albumin followed an opposite trend. Tied male dromedaries had the lowest (*p* < 0.001) concentrations of blood serum glucose and cholesterol, followed by exercised and fenced male dromedaries. The interaction analysis between animal social contact and movement control revealed that the single-tied and group-tied dromedaries had the highest (*p* < 0.001) concentrations of blood serum total protein and globulin, followed by those in the single-exercised and group-exercised dromedaries, and the lowest values were in the single-fenced and group-fenced dromedaries. The single-exercised and group-exercised dromedaries had the highest (*p* < 0.001) concentrations of blood serum total protein and globulin, followed by those in the single-fenced and group-fenced dromedaries, and the lowest values were in the single-tied and group-tied dromedaries.

### 3.4. Effect on Liver and Kidney Function Indicators and Redox Status

The effects of animal social contact (single or group), movement control (tied, fenced or exercised) and their interaction on liver (AST and ALT) and kidney (creatinine and urea) function indicators and redox status (MDA, TAC and NO) indicators of male dromedaries during the rutting season are presented in Table 5. Among the blood serum variables analyzed, animal social contact significantly affected concentrations of blood serum AST and TAC; single-housed male dromedaries had higher AST and TAC concentrations than group-housed male camel. Movement-control method significantly affected concentrations of blood serum AST, ALT, MDA, TAC and NO. Compared to fenced and exercised male dromedaries, tied male dromedaries had the lowest significant concentrations of AST, ALT, MDA and, NO and the highest (*p* < 0.001) concentrations of TAC. The Single-fenced, Single-exercised, group-fenced and group-exercised dromedaries had higher (*p* < 0.001) concentrations of blood serum AST, ALT and NO and lower (*p* = 0.011) concentrations of TAC than those in the single-tied and group-tied dromedaries.

### 3.5. Effect on Hormone Levels and Mineral Balance

The effects of animal social contact (single or group), movement control (tied, fenced or exercised) and their interaction on concentrations of blood serum hormones and minerals of male dromedaries during the rutting season are presented in Table 6. Animal social contact significantly affected the concentration of blood serum cortisol. Single-housed male dromedaries had higher (*p* = 0.004) cortisol concentrations than group-housed male dromedaries. Movement-control method significantly affected concentrations of blood serum cortisol, testosterone and T3. Tied male dromedaries had the highest significant concentrations of blood serum cortisol and T3; while, fenced male dromedaries had the highest concentrations of blood serum testosterone. The single-tied and group-tied dromedaries had highest (*p* < 0.001) concentrations of blood serum cortisol and T3, followed by those in the single-exercised and group-exercised dromedaries while those in the Single-fenced and group-fenced dromedaries had the lowest values. The group-fenced dromedaries had the highest concentrations of blood serum testosterone, whereas the group-exercised dromedaries had the lowest values, and male dromedaries in the other groups had intermediate values. Serum mineral concentrations were not affected by animal social contact, movement control and their interaction. Except the significant increases in magnesium concentrations (*p* = 0.005) in the fenced and exercised groups and potassium concentrations (*p* = 0.026) in fenced group. The group-fenced dromedaries had higher (*p* = 0.020) concentrations of serum magnesium than in the single-tied group.

## 4. Discussion

In this study, social contact of male dromedaries resulted in significant increases in maintenance and posture behaviors and sexual activity-related behaviors. Furthermore, longer duration was devoted for lying and standing behaviors in single-housed animals, which supports this hypothesis. This behavior may reflect the boredom status of single-housed animals due to social isolation, which could be considered one of the stressful factors as indicated by high concentration of serum cortisol in these animals.

Effects of movement control may indicate that allowing locomotion activity and exercise for male dromedaries is a suitable management measure for male dromedaries during the rutting season. Fatnassi et al. [15] suggested that the freedom to move and to express social behavior as “behavioral needs” confer adequate wellbeing for camel reared for semen collection. In addition, Padalino et al. [17] found that male dromedaries that were reared with one hour free for walking around and thirty minutes of the exposition to the female herd reduced the occurrence of the stereotypies. McDonnel [21] stated that placing the stallion under the natural day light cycle with chances to practice exercise can improve reproductive performance and overcome specific breeding problems. In context, Freire et al. [22] found that one hour or regular exercise in a paddock has positive effects on horse welfare. Overall, exercise presents an important management practice for reducing tedium and weariness due to confinement stress and social isolation [23]. These positive effects of allowing locomotion activity on male camel behavior could be easily confirmed in our study as all male dromedaries fenced or exercised expressed better maintenance, locomotion and sexual behaviors, regardless they were individually or collectively housed.

In this study, rather animal social contact, movement control seems to be the major factor that controls metabolism and hormonal balance of animals. Male dromedaries allowed to practice locomotion activity, fenced or exercised, had elevated transaminase activities and decreased redox status. These findings are somewhat expected, as physical activity was found to be associated with elevated liver ALT and AST levels [24,25,26] and oxidative stress. The elevations in these metabolites could be attributed to the muscular activity and energy utilization during locomotion activity [27].

Interestingly, male dromedaries allowed to practice locomotion activity, fenced or exercised, had high concentrations of circulating energy yielding nutrients, glucose and cholesterol. This result may be attributed to the improved feed digestion and nutrients absorption as a result of increased rumination frequency and duration. In addition, these animals had low concentrations of blood serum cortisol and T3 and increased concentrations of blood serum testosterone. Cortisol was identified as the main indicator of the stress because their level drastically rises due to stressful situations. In this study, it could be clearly indicated that tied and single housed male dromedaries suffered from stress due to these housing systems. These results are in accordance with those obtained in previous studies [15,28] in which male dromedaries housed in single stalls for 24 h expressed discomfort signs and had elevated cortisol concentrations. The decreased blood serum concentrations of testosterone observed in this study in tied male dromedaries could be ascribed to the adverse relationship between cortisol and testosterone. Increased cortisol concentrations negatively affected testosterone production by Leydig cells [29]. Tied male dromedaries had higher T3 concentrations than those allowed to practice locomotion activity. T3 is a metabolic hormone employed as an indicator of metabolic activity in animals [30]. Thus, reduced concentrations of blood serum T3 in male dromedaries allowed to practice locomotion activity may be one of adaptive mechanisms developed as an attempt to reduce metabolic rate and thus excess metabolic heat production by active animals [25].

## 5. Conclusions

Animal social interaction is of particular importance for maintaining physical, psychological and sexual behaviors. Allowing locomotion activity for captive animals improves their metabolic status and decreases captive stress effects. Fenced and exercised male dromedaries had significantly higher concentrations of blood serum transaminases, free radicals, glucose, cholesterol and testosterone and significantly lower concentrations of cortisol and triiodothyronine than tied male dromedaries. Housing systems that guarantee social interaction and physical activity are being the most suitable housing management systems for male dromedaries during the rutting season.

## Figures and Tables

**Table 1:** Behavioral ethogram of dromedaries.

	Behavior	Definition
Behavior state	Standing	Camel stands in inactive upright posture on all four feet with no movement.
	Lying	Camel body contacts the ground or camel sits in sternal recumbency.
	Walking	Camel does more than 2 complete steps.
	Ruminating	Camel regurgitates a bolus of food into the mouth, then rechews and re-swallows it while standing or lying down.
Sexual behavior events	Teeth grinding	Camel moves lower jaw left and right with mouth closed, grinding the teeth and producing a typical squeaking/whistling sound.
	Blathering	Camel emits typical gurgling and roaring sounds
	Dulla extruding	Camel exteriorizes the soft palate, usually known as the “dulla’’.
	Tail flapping	Camel holds tail under the prepuce, then beats it up and down 4 to 5 times, spreading urine over the croup and surrounding areas.
	Leg opening	Camel splays hind legs until they form an angle of at least 45 degrees.
	Urinating	Camel eliminates urine.

**Table 2:** Effects of social contact, movement control and their interaction on frequency (n/20 min) and duration (sec/20 min) of maintenance and posture behaviors of male dromedaries during the rutting season.

Factors	Variable							
	Maintenance		Posture					
	Ruminating		Lying		Standing		Walking	
	Frequency	Duration	Frequency	Duration	Frequency	Duration	Frequency	Duration
Social contact								
Single	6.97 ^b^	78.50 ^b^	2.44 ^b^	139.08 ^a^	5.89 ^b^	118.75 ^a^	3.33 ^b^	54.89 ^b^
Group	12.86 ^a^	91.11 ^a^	5.55 ^a^	114.66 ^b^	8.00 ^a^	101.42 ^b^	6.67 ^a^	75.83 ^a^
SEM	0.463	0.979	0.270	1.39	0.412	1.60	0.435	1.17
*p* value	<0.001	<0.001	<0.001	<0.001	<0.001	<0.001	<0.001	<0.001
Movement control								
Tied	7.83 ^b^	79.00 ^c^	6.50 ^a^	154.63 ^a^	3.50 ^c^	88.75 ^c^	1.50 ^c^	50.25 ^b^
Fenced	9.25 ^ab^	85.25 ^b^	3.33 ^b^	115.00 ^b^	7.67 ^b^	116.00 ^b^	5.00 ^b^	62.33 ^b^
Exercised	12.66 ^a^	90.16 ^a^	2.17 ^c^	111.00 ^b^	9.67 ^a^	125.50 ^a^	8.50 ^a^	73.50 ^a^
SEM	0.513	1.08	0.299	1.56	0.456	1.77	0.482	1.30
*p* value	<0.001	<0.001	<0.001	<0.001	<0.001	<0.001	<0.001	<0.001
Social contact × movement control								
Single-tied	7.25 ^cd^	80.50 ^c^	6.00 ^b^	161.25 ^a^	5.03 ^c^	92.25 ^d^	1.21 ^c^	45.00 ^c^
Single-fenced	5.33 ^d^	73.73 ^d^	2.33 ^cd^	130.00 ^c^	8.07 ^b^	127.00 ^b^	4.12 ^bc^	57.67 ^b^
Single-exercised	8.33 ^c^	82.02 ^c^	1.10 ^d^	126.12 ^c^	11.00 ^a^	137.00 ^a^	5.04 ^b^	62.71 ^b^
Group-tied	11.25 ^ab^	90.00 ^b^	9.00 ^a^	148.00 ^b^	2.00 ^d^	85.25 ^d^	2.93 ^c^	55.50 ^b^
Group-fenced	10.33 ^b^	85.17 ^c^	4.33 ^bc^	100.07 ^d^	7.33 ^bc^	105.00 ^c^	6.50 ^b^	87.09 ^a^
Group-exercised	17.00 ^a^	98.33 ^a^	3.33 ^c^	96.25 ^d^	8.33 ^b^	114.00 ^c^	12.00 ^a^	85.10 ^a^
SEM	0.70	1.5	0.54	2.5	0.63	2.69	0.79	2.1
*p* value	0.015	0.021	0.027	0.003	0.029	0.018	0.003	0.001

Means ± SEM in each column with no common superscript letter differ significantly (*p* < 0.05).

**Table 3:** Effects of social contact, movement control and their interaction on sexual behavior of male dromedaries during the rutting season.

Factors	Sexual Behavior Parameters					
	Teeth Grinding	Leg Opening	Tail Flapping	Dulla Extruding	Blathering	Urinating
Social contact						
Single	5.33 ^b^	3.56 ^b^	2.33 ^b^	2.67 ^b^	2.53 ^b^	5.08 ^b^
Group	7.00 ^a^	5.22 ^a^	3.33 ^a^	4.67 ^a^	3.75 ^a^	6.19 ^a^
SEM	0.435	0.393	0.381	0.362	0.313	0.350
*p* value	<0.001	<0.001	<0.001	0.009	0.002	<0.001
Movement control						
Tied	2.50 ^c^	1.50 ^c^	1.00 ^c^	2.50 ^b^	1.75 ^b^	2.75 ^b^
Fenced	7.00 ^b^	5.00 ^b^	2.50 ^b^	4.00 ^a^	3.33 ^a^	6.83 ^a^
Exercised	9.00 ^a^	6.67 ^a^	5.00 ^a^	4.50 ^a^	4.33 ^a^	7.33 ^a^
SEM	0.482	0.504	0.422	0.401	0.347	0.387
*p* value	0.017	0.004	0.005	0.001	0.01	0.041
Social contact × movement control						
Single-tied	2.23 ^c^	1.05 ^c^	1.20 ^c^	2.50	1.25	2.25 ^c^
Single-fenced	6.47 ^b^	4.06 ^b^	2.00 ^bc^	3.17	2.33	7.00 ^b^
Single-exercised	8.05 ^ab^	5.67 ^ab^	4.2 ^ab^	3.00	4.00	6.00 ^b^
Group-tied	3.21 ^c^	2.00 ^c^	1.07 ^c^	3.20	2.25	3.25 ^c^
Group-fenced	8.20 ^ab^	6.00 ^ab^	3.00 ^ab^	5.52	4.33	6.67 ^b^
Group-exercised	10.42 ^a^	7.67 ^a^	6.20 ^a^	6.00	4.67	8.67 ^a^
SEM	0.68	0.58	0.60	0.54	0.50	0.57
*p* value	0.029	0.023	0.033	0.255	0.603	0.039

Means ± SEM in each column with no common superscript letter differ significantly (*p* < 0.05).

**Table 4:** Effects of social contact, movement control and their interaction on concentrations of blood serum major proteins and energy-yielding metabolites of male dromedaries during the rutting season.

Factors	Variables					
	Major Blood Serum Proteins (g/dL)			Energy-Yielding Metabolites (mg/dL)		
	Total Protein	Albumin	Globulin	Glucose	Cholesterol	Triglycerides
Social contact						
Single	6.91	3.14	3.69	82.83	53.55	54.67
Group	6.80	3.14	3.80	82.30	53.96	55.74
SEM	0.114	0.149	0.416	1.69	0.831	1.11
*p* value	0.579	0.989	0.842	0.828	0.735	0.502
Movement control						
Tied	7.26 ^a^	2.65 ^b^	4.91 ^a^	68.38 ^c^	39.45 ^c^	54.75
Fenced	5.95 ^c^	3.36 ^a^	2.59 ^b^	82.38 ^b^	55.40 ^b^	52.83
Exercised	6.75 ^b^	3.41 ^a^	3.85 ^b^	96.95 ^a^	66.43 ^a^	58.03
SEM	0.146	0.18	0.431	1.87	1.06	1.41
*p* value	<0.001	0.014	<0.001	<0.001	<0.001	0.060
Social contact × movement control						
Single-tied	7.51 ^a^	2.73	4.78 ^a^	68.51 ^c^	39.00 ^c^	54.00
Single-fenced	5.91 ^c^	3.28	2.63 ^c^	81.67 ^b^	52.00 ^b^	54.67
Single-exercised	6.70 ^b^	3.40	3.30 ^b^	98.33 ^a^	67.00 ^a^	58.00
Group-tied	7.61 ^a^	2.58	5.04 ^a^	68.25 ^c^	39.88 ^c^	55.50
Group-fenced	5.98 ^c^	3.4	2.55 ^c^	83.10 ^b^	56.13 ^b^	53.67
Group-exercised	6.80 ^b^	3.4	3.40 ^b^	95.57 ^a^	65.88 ^a^	58.07
SEM	0.179	0.270	0.228	3.05	1.51	1.73
*p* value	<0.001	0.835	<0.001	<0.001	<0.001	0.905

Means ± SEM in each column with no common superscript letter differ significantly (*p* < 0.05).

**Table 5:** Effects of social contact, movement control and their interaction on liver and kidney function indicators and redox status indicators of male dromedaries during the rutting season.

Factor	Variables						
	Liver and Kidney Function Indicators				Redox Status Indicators		
	AST (U/L)	ALT (U/L)	Creatinine (mg/dL)	Urea (mg/dL)	MDA (nmol/mL)	TAC (mM/L)	NO (µmol/L)
Social contact							
Single	23.00 ^a^	20.24	1.46	13.77	1.63	1.69 ^a^	70.50
Group	21.06 ^b^	21.39	1.33	13.45	1.97	1.41 ^b^	73.56
SEM	3.65	2.36	0.021	0.675	0.129	0.058	1.27
*p* value	0.003	0.095	0.067	0.737	0.074	0.004	0.093
Movement control							
Tied	12.99 ^c^	15.99 ^c^	1.48	14.79	1.48 ^b^	1.84 ^a^	62.21 ^c^
Fenced	22.34 ^b^	22.22 ^b^	1.39	13.82	2.10 ^a^	1.43 ^b^	72.00 ^b^
Exercised	30.66 ^a^	25.83 ^a^	1.28	12.22	1.93 ^ab^	1.63 ^ab^	85.17 ^a^
SEM	4.04	1.87	0.059	0.728	0.169	0.075	1.52
*p* value	<0.001	<0.001	0.094	0.103	0.031	<0.001	<0.001
Social contact × movement control							
Single-tied	10.87 ^c^	15.9 ^d^	1.58	14.98	1.35	1.98 ^a^	60.50 ^c^
Single-fenced	12.41 ^bc^	21.30 ^c^	1.47	14.00	1.87	1.57 ^bc^	70.33 ^b^
Single-exercised	13.27^ab^	25.00 ^ab^	1.30	12.33	1.77	1.53 ^bc^	84.00 ^a^
Group-tied	12.77 ^bc^	16.14 ^d^	1.38	14.61	1.61	1.70 ^b^	63.91 ^c^
Group-fenced	15.15 ^a^	23.10 ^bc^	1.31	13.63	2.33	1.30 ^cd^	73.67 ^b^
Group-exercised	15.53 ^a^	26.67 ^a^	1.27	12.11	2.00	1.21 ^d^	86.33 ^a^
SEM	5.72	0.89	0.07	1.19	0.23	0.11	1.86
*p* value	<0.001	<0.001	0.093	0.997	0.076	0.011	<0.001

AST—aminase; ALT—alanine transaminase; TAC—total antioxidant capacity; MDA—malondialdehyde; NO—nitric oxide; means ± SEM in each column with no common superscript letter differ significantly (*p* < 0.05).

**Table 6:** Effects of social contact, movement control and their interaction on concentrations of blood serum hormones and minerals of male dromedaries during the rutting season.

Factor	Variables						
	Hormones			Minerals			
	Cortisol (nmol/L)	Testosterone (ng/mL)	Triiodothyronine (ng/mL)	Calcium (mg/dL)	Phosphorous (mg/dL)	Magnesium (mg/dL)	Potassium (mg/dL)
Social contact							
Single	66.89 ^a^	2.05	1.09	2.42	2.27	0.914	3.67
Group	62.54 ^b^	2.41	1.08	2.54	2.31	1.04	3.87
SEM	0.707	0.190	0.044	0.078	0.033	0.051	0.017
*p* value	0.004	0.113	0.728	0.361	0.765	0.106	0.162
Movement control							
Tied	89.81 ^a^	1.69 ^b^	2.03 ^a^	2.55	2.31	0.796 ^b^	3.55 ^b^
Fenced	44.12 ^c^	2.91 ^a^	1.68 ^b^	2.40	2.29	1.13 ^a^	4.05 ^a^
Exercised	53.52 ^b^	2.09 ^ab^	1.91 ^b^	2.47	2.25	0.998 ^a^	3.88 ^ab^
SEM	0.783	0.116	0.056	0.078	0.033	0.064	0.091
*p* value	<0.001	0.002	<0.001	0.613	0.807	0.005	0.026
Social contact × movement control							
Single-tied	91.00 ^a^	1.95 ^bc^	2.03 ^a^	2.48	2.25	0.78 ^c^	3.48
Single-fenced	46.63 ^c^	2.67 ^ab^	1.68 ^c^	2.37	2.30	1.07 ^ab^	3.90
Single-exercised	55.00 ^b^	1.53 ^c^	1.90 ^b^	2.40	2.28	0.90 ^bc^	3.70
Group-tied	88.63 ^a^	2.23 ^bc^	2.05 ^a^	2.63	2.38	0.82 ^bc^	3.62
Group-fenced	41.60 ^c^	3.15 ^a^	1.70 ^c^	2.43	2.29	1.20 ^a^	4.20
Group-exercised	52.03 ^b^	1.85 ^bc^	1.92 ^b^	2.55	2.23	1.10 ^ab^	3.86
SEM	1.07	0.24	0.06	0.14	0.09	0.07	0.15
*p* value	<0.001	0.009	<0.001	0.953	0.668	0.020	0.091

Means ± SEM in each column with no common superscript letter differ significantly (*p* < 0.05).
